# Technique of extracting a compression intramedullary nail that preserves knee arthrodesis

**DOI:** 10.3109/17453674.2011.584207

**Published:** 2011-07-08

**Authors:** Terence P Murphy, Kevin J Mulhall

**Affiliations:** Department of Orthopaedic Surgery, Mater Misericordiae University Hospital, Dublin, Ireland

Intramedullary compression nails have recently been developed for knee arthrodesis. These implants have given fusion in 90–100% of cases regardless of the initial diagnosis or experience of the user (Domingo et al. 2004, McQueen et al. 2006, De et al. 2008). However, many clinicians have been reluctant to use this technique due to the perceived difficulty in or impossibility of removing these nails without disrupting the fusion, to allow the management of associated complications such as infection.

We present the case of a patient who required removal of a compression intramedullary fusion nail for recurrent infection, in order to describe an easily performed extraction technique that does not disrupt the arthrodesis and that allows definitive management of infection.

A 48-year-old man was referred with a painful failed knee arthrodesis that had originally been attempted for failed primary knee arthroplasty secondary to infection. There were no clinical or hematological signs of residual infection, so we proceeded to attempt fusion with an intramedullary compression nail (Wichita Fusion Nail; Stryker). He developed recurrent infection, which was managed with appropriate antibiotics until solid fusion was achieved. We then proceeded to remove the nail 8 months after the fusion procedure and to manage the infection more definitively (Figure 1).

**Figure 1.**
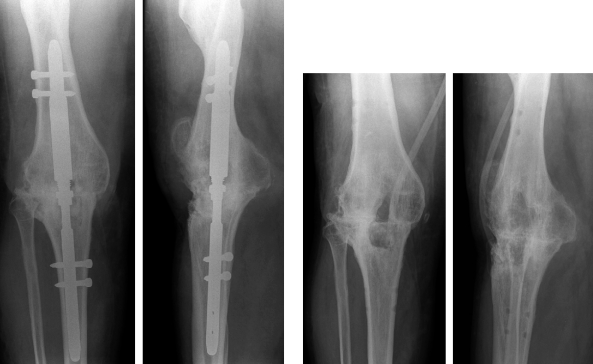
A. 8 months after fusion. B. 1 month after nail removal.

## Surgical technique

**Figure 2.**
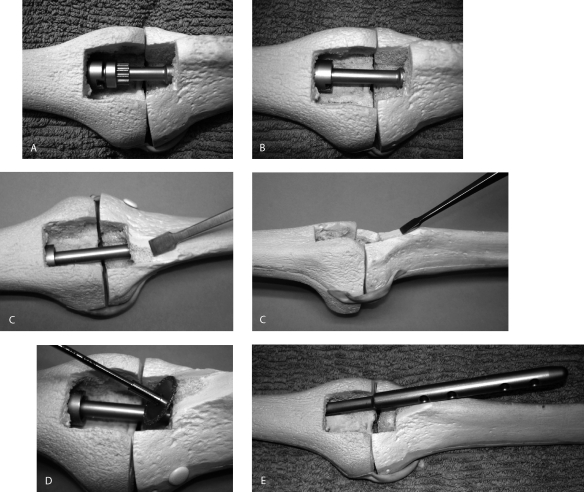
A. Reopening of osteotomy of the original anterior bony window. B. Removal of the locking nut. C. Direction of anterior sloping extension of anterior window osteotomy in the tibia to allow extraction. D. Use of a high-speed power metal cutting disc to cut the nail at the appropriate angle. E. Extraction of the distal tibial portion after telescoping into femoral canal and then angling out.

The patient is positioned supine with a high thigh tourniquet applied. Access is gained through the incision used for nail insertion. The 4 locking screws are removed via previous stab incisions. The site of the previous anterior window osteotomy is reopened (Figure 2A). Easy access is thus obtained to the compression screw which is then removed (Figure 2B). With the locking screws and compression screw removed, it is then possible to slide the intramedullary nail up and down the canal. The anterior window osteotomy is extended by adding an angled anterior component to the tibial end of the osteotomy window, taking care not to remove the tibial tuberosity (Figure 2C). The tibial component of the nail can also telescope within the femoral component, and this should be performed to the maximal extent. This shortens the construct as much as possible. The implant is sectioned through the tibial component just below the femoral component, as the femoral side is shorter (Figure 2D). A power metal cutting disc (Midas Rex; Medtronic, TX) is used to obtain an angled cut of the implant. This cut must be angled enough to allow the femoral part of the nail to be maneuvered out. Once removed, the tibial component can then be slid up the femoral canal and removed in a similar fashion to the femoral side (Figure 2E).

In the presence of infection—as in this case—thorough debridement, canal reaming, copious lavage, and possible application of special dressings (as desired) is possible. The patient was initially allowed partial weight bearing in a brace for 6 weeks to protect the fusion. The fusion was intact and the patient was walking without pain or any aids at the 1-year follow-up, with no further evidence of infection.

We feel that the technique described here is simple and repeatable, and should alleviate some of the existing concerns with use of these devices.
